# Metabolomics-Based Machine Learning Diagnostics of Post-Acute Sequelae of SARS-CoV-2 Infection

**DOI:** 10.3390/metabo15120801

**Published:** 2025-12-17

**Authors:** Ethan Cai, Valentina L. Kouznetsova, Igor F. Tsigelny

**Affiliations:** 1 REHS Program, San Diego Supercomputer Center, University of California San Diego, San Diego, CA 92093, USA; ethanc8858@gmail.com; 2 San Diego Supercomputer Center, University of California San Diego, San Diego, CA 92093, USA; vkouznetsova@ucsd.edu; 3 CureScience Institute, San Diego, CA 92121, USA; 4 BiAna Institute, San Diego, CA 92117, USA; 5 Department of Neurosciences, University of California San Diego, San Diego, CA 92093, USA

**Keywords:** PASC, Long COVID, ME/CFS, POTS, IBS, fibromyalgia, machine learning, metabolites

## Abstract

**Background:** COVID-19 has taken millions of lives and continues to affect people worldwide. Post-Acute Sequelae of SARS-CoV-2 Infection (also known as Post-Acute Sequelae of COVID-19 (PASC) or more commonly, Long COVID) occurs in the aftermath of COVID-19 and is poorly understood despite its widespread effects. **Methods:** We created a machine-learning model that distinguishes PASC from PASC-similar diseases. The model was trained to recognize PASC-dysregulated metabolites (*p* ≤ 0.05) using molecular descriptors. **Results:** Our multi-layer perceptron model accurately recognizes PASC-dysregulated metabolites in the independent testing set, with an AUC-ROC of 0.8991, and differentiates PASC from myalgic encephalomyelitis/chronic fatigue syndrome (ME/CFS), Lyme disease, postural orthostatic tachycardia syndrome (POTS), and irritable bowel syndrome (IBS). However, it was unable to differentiate fibromyalgia (FM) from PASC. **Conclusions:** By creating and testing models pairwise on each of these diseases, we elucidated the unique strength of the similarity between FM and PASC relative to other PASC-similar diseases. Our approach is unique to PASC diagnosis, and our use of molecular descriptors enables our model to work with any metabolite where molecular descriptors can be identified, as these descriptors can be generated and compared for any metabolite. Our study presents a novel approach to PASC diagnosis that partially circumvents the lengthy process of exclusion, potentially facilitating faster interventions and improved patient outcomes.

## 1. Introduction

COVID-19 is a highly contagious disease caused by the novel coronavirus, SARS-CoV-2, characterized by symptoms such as fever, chills, and sore throat, which typically appear within a week of exposure and last up to two weeks. It has had a profound and unprecedented impact on the modern world. According to the World Health Organization (WHO), as of 28 September 2025, a total of 778.7 million cases and 7.1 million deaths have been recorded globally [1].

Due to the widespread urgency of the COVID-19 pandemic, its diagnosis, causes, and treatment have been thoroughly studied and investigated. In contrast, Post-Acute Sequelae of SARS-CoV-2 Infection (PASC), a condition that follows COVID-19, remains poorly understood, despite its widespread impact [2].

The WHO defines Long COVID as “the continuation or development of new symptoms three months after the initial SARS-CoV-2 infection, with these symptoms lasting for at least two months with no other explanation.” Symptoms and severity may differ from those in the acute phase, and more than 200 symptoms have been reported across multiple bodily systems, with fatigue, cognitive dysfunction, and shortness of breath among the most common [2,3]. Terminology for the condition varies; many sources use Long COVID, post-COVID-19 syndrome (PCS), and PASC interchangeably [4], while others distinguish between them. The WHO also uses the term post-COVID-19 condition (PCC), and the Researching COVID to Enhance Recovery (RECOVER) Initiative [5] defines it as Post-Acute Sequelae of SARS-CoV-2 Infection. In this study, we use “Long COVID” and “PASC” interchangeably, following the definition set out by the WHO [2].

Currently, Long COVID remains poorly understood and defined, in terms of terminology prevalence, occurrence, and biomarkers. The etiology of PASC remains unclear, although several hypotheses have been proposed. One suggestion is that PASC is caused by remnants of the coronavirus persisting in some organs and continuing to provoke an immune response even after the immune system has eliminated the virus, leading to chronic inflammation and tissue damage [6]. Another proposed hypothesis claims PASC sources from the reactivation of latent viruses, such as the Epstein-Barr virus (EBV) or the suggestion that PASC sources may stem from the reactivation of latent viruses, such as the Epstein-Barr virus (EBV) or Human Herpesvirus 6 (HHV6) [6,7]. It has also been proposed that PASC is caused by a combination of several coexisting factors, rather than a single factor.

As of January 2025, there is no formal diagnostic test for PASC [8]. Diagnosing PASC is particularly challenging because its symptoms are inconsistent and may manifest in different ways across multiple organs, making it difficult to identify and differentiate from other conditions [7,8,9]. The RECOVER study found that these symptoms change, disappear, and recur unpredictably [5]. Deer et al. reported that the symptoms of PASC are “extremely heterogeneous” and that among 15 studies that identified 55 symptoms, none were consistently present in every study [10]. The complexity of the disease and the lack of specific biomarkers make diagnosis cumbersome [11]. Statistics and understandings of this condition vary widely, with the estimated percentage of COVID-19 patients who developed PASC ranging from 10% to 60% depending on the source.

Another complication with PASC diagnosis is the similarity of symptoms between PASC and other diseases. For instance, fibromyalgia (FM) and myalgic encephalomyelitis/chronic fatigue syndrome (ME/CFS) share many overlapping symptoms. One study found that “one-third of long COVID patients met the diagnostic criteria for CFS/ME at six months after the initial infection” [12] and that “Two different surveys found that 20–31% of long COVID patients met classification criteria for FM more than six months after COVID-19” [12]. There is also evidence that links PASC, FM, ME/CFS, and, in addition, irritable bowel syndrome (IBS), finding them to be associated with the reactivation of endogenous viruses, particularly the latent EBV [12]. Furthermore, other studies have also identified significant similarities in symptoms causing confusion in distinguishing between PASC and other illnesses, including postural orthostatic tachycardia syndrome (POTS) and Lyme disease [13,14].

Currently, PASC is often diagnosed through a lengthy process of exclusion that relies heavily on observing a patient’s medical history. Most proposed diagnostic processes start by excluding a list of similar medical conditions, analyzing medical history, and then observing common symptoms of PASC, such as dyspnea or anosmia/dysgeusia. Depending on the case, special tests such as X-rays or pulmonary function assessments may be employed as needed [15,16].

To address the complexity and inefficiency of such diagnostics, some researchers have turned to AI-based tools to accelerate the process. Several studies use traditional exclusion methods, supported by analyzing patients’ past medical history and tracking the development of symptoms over time to diagnose long-term COVID-19 [17]. Others bypass exclusion entirely. As an example, Buonsenso et al. applied proteomics to predict long COVID with 93% accuracy [18].

Our aim is to evaluate the application of a molecular descriptor-based approach to distinguish between PASC and PASC-similar diseases, thereby improving the efficiency of PASC diagnosis. Specifically, we propose a machine learning (ML) system to develop a diagnostic system for PASC that utilizes molecular descriptors of dysregulated plasma metabolites to distinguish PASC patients from both healthy individuals and those with PASC-like symptoms, thereby reducing reliance on exclusion-based methods. We hypothesize that an ML model trained to identify PASC-associated plasma metabolites can aid in the elucidation of patients’ diagnoses. To improve specificity, we tested our model against other diseases often misdiagnosed as PASC, aiming to distinguish between PASC and PASC-like diseases.

This approach, using molecular descriptors, is novel in the context of PASC but has been applied to other diseases. Yao et al. utilized molecular descriptors (calculated using PaDEL-Descriptor software, version 2.21 [19]) of urinary metabolites to diagnose ovarian cancer, employing a random forest model that yields a specificity of 0.86 and sensitivity of 0.97 with 10-fold cross-validation [20]. Yang et al. employed molecular descriptors (calculated using ChemDes software, version 3.0 [21]) of serum metabolites to identify colorectal cancer, achieving up to 95.21% 10-fold cross-validated accuracy with a bagging algorithm [22].

## 2. Methods

### 2.1. Approach Overview

**Figure 1:**
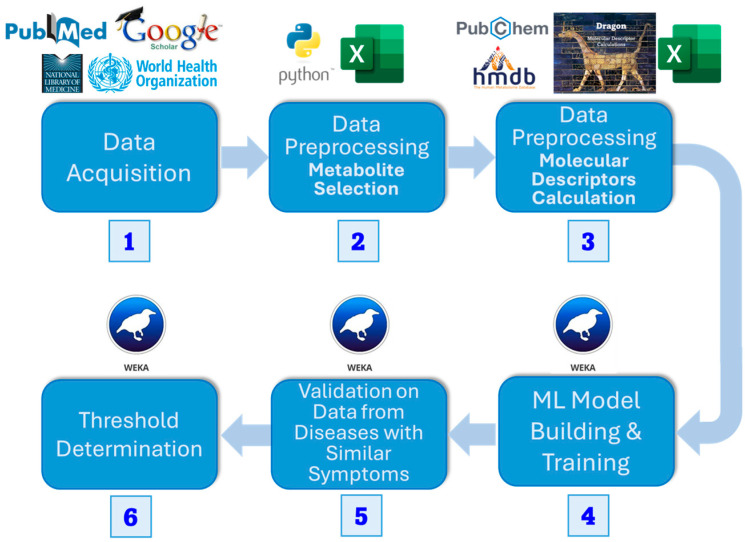
Flowchart of methods. We (1) acquired datasets for PASC patients, (2) found dysregulated metabolites using a *t*-test, and (3) calculated the molecular descriptors for the metabolite datasets. Then we (4) built an ML model and (5) tested it on diseases with similar symptoms (6) to define a threshold for dysregulated metabolites classified as PASC to classify the disease itself.

The method’s flowchart is represented in Figure 1.

We collected plasma metabolic data from open sources, including PubMed, Google Scholar, and the National Library of Medicine. Significant metabolites were selected using univariate *t*-test analysis (*p* ≤ 0.05). Then, we retrieved the same number of random metabolites from the Human Metabolite Database (HMDB) [23], preprocessed them in the same manner, and combined them with the dysregulated metabolites. Next, we calculated a set of molecular descriptors using the e-Dragon software, version 5.4 [24], and then performed dimensionality reduction using CfsSubsetEval and BestFirst search from the Waikato Environment for Knowledge Analysis (WEKA) [25]. Utilizing the prepared dataset, we trained several machine learning algorithms in Python, version 3.13.5, as implemented by Scikit-Learn, version 1.7.2 [26], using 5-fold cross-validation. After selecting the best ones, we validated them using metabolic data from diseases with PASC-like symptoms. We then determined a threshold to convert the classifier’s output score into a final, definitive class prediction, striking a balance between sensitivity and specificity.

### 2.2. Data Collection and Preprocessing

The plasma PASC metabolites datasets used in this study for model training and testing were all obtained from publicly accessible, peer-reviewed sources, namely from PubMed and the National Library of Medicine [4,27]. Both sets contained metabolites of healthy individuals and PASC patients. We used this data to determine the metabolites dysregulated by PASC by running a two-tailed two-sample *t*-test, assuming unequal variance, and selecting metabolites with a *p*-value ≤ 0.05. Because we are looking for any metabolites that are significantly dysregulated, regardless of the degree of such dysregulation, we use a *t*-test to examine metabolite dysregulation in relation to the rest of each dataset. Demographic data for the training and testing sets are shown in Table 1. Our analysis identified 123 and 36 dysregulated metabolites from the training and testing sets, respectively. We also utilized dysregulated metabolite sets associated with other diseases to evaluate the model’s ability to distinguish between PASC and PASC-like diseases. The diseases known to have similar symptomology to PASC are FM, ME/CFS, Lyme disease, POTS, and IBS [28,29,30,31,32]. To utilize these metabolites for training the model to predict whether dysregulation was due to PASC, we randomly selected from HMDB 5.0 [23] the same number of metabolites used as a negative control to PASC-dysregulated metabolites. In doing so, we balance our dataset, thereby preventing model variance caused by dataset imbalance. In the case that PASC-related metabolites from our selected list appear in the random set, those are excluded. To confirm that the selection of negative metabolites has a minimal impact on model performance, we performed a 90/10% stratified split on the training dataset. Then, for 100 bootstrapping iterations, we created a new negative control set of the same size by resampling the original 90% split of negative controls with replacement. By concatenating this set with the original 90% split of PASC metabolites, we trained a random forest model and tested it on the independent testing set. Indeed, the standard deviation of cross-validated accuracy across 100 bootstrapped runs was 0.0109, indicating minimal variance due to randomness in the selection of negative controls.

### 2.3. Molecular Descriptor Calculation

We first converted each metabolite into its Simplified Molecular Input Line Entry System (SMILES) [33] form using HMDB [23], PubChem [34], and the NCI/CADD Group’s CACTUS (CADD Group Chemoinformatic Tools and User Services) API [35] to streamline this process. Then, we used e-Dragon Software [36] to generate 4179 molecular descriptors from each metabolite. To reduce noise and prevent overfitting, we utilized WEKA’s version 3.9.6 correlation-based feature selection (CfsSubsetEval) algorithm, which resulted in 28 descriptors from 4179 [25]. This algorithm selects a subset of molecular descriptors such that the correlation between the molecular descriptors and the presence of PASC is maximized, while minimizing the correlation between each of the molecular descriptors within the subset, thereby reducing redundancy.

### 2.4. Machine Learning Model Training

Using the molecular descriptors of the PASC-dysregulated and randomly selected metabolites, we applied several machine learning classifiers (as implemented by Scikit-Learn [26]) to create ML models that utilize the molecular descriptors of each classifier to predict whether the metabolite was dysregulated by PASC. We tested the following classifiers: multi-layer perceptron (MLP), random forest (RF), logistic regression (LR), support vector machine (SVM), and gradient boosting (GB). For each model, we employed hyperparameter selection using grid search along with stratified 5-fold cross-validation. The core statistic used to measure model performance was the AUC-ROC, which was evaluated using repeated 5-fold cross-validation. We report results with a mean AUC-ROC and the corresponding 95% confidence interval. Here, we use the area under the receiver operating characteristic curve (AUC-ROC) because it gives a broad summary of model performance across all possible classification thresholds.

### 2.5. Validation on Independent Testing Sets and Other Diseases

We selected the top-performing models and tested them against dysregulated metabolite sets for an independent PASC testing set. We then tested these top-performing models against dysregulated metabolite sets for an independent PASC testing set, along with diseases that are often confused with PASC. For each disease set, the proportion of metabolites classified as PASC-dysregulated was recorded, along with the mean and confidence interval. The confidence interval was obtained by bootstrapping the testing set, repeating the resampling process 500 times, and calculating the 95% confidence interval from the middle. Note that, because the model is trained to flag each individual metabolite as PASC-dysregulated using each metabolite’s 28 consistent descriptors, the asymmetry in the number of metabolites in the training and testing sets does not impact performance

### 2.6. Threshold Determination

A crucial aspect of this study is the diagnosis of PASC and differentiating it from other disorders with similar symptoms. For this purpose, we used the previously calculated confidence intervals to determine a threshold that maximizes the separation between the confidence intervals of percent metabolites classified as PASC and those of similar diseases. When the proportion of dysregulated metabolites classified by the model as being PASC-dysregulated exceeds this threshold, a confident PASC diagnosis can be suggested.

## 3. Results

We identified 123 PASC-dysregulated metabolites (*p* ≤ 0.05) and combined them with 123 randomly selected metabolites from HMDB 5.0 to form a 246-metabolite training set. For each metabolite, we calculated 4179 molecular descriptors using e-Dragon, and attribute selection narrowed the feature space down to a set of 28 meaningful molecular descriptors. A list of the selected descriptors is included in the Supplementary Materials.

**Figure 2:**
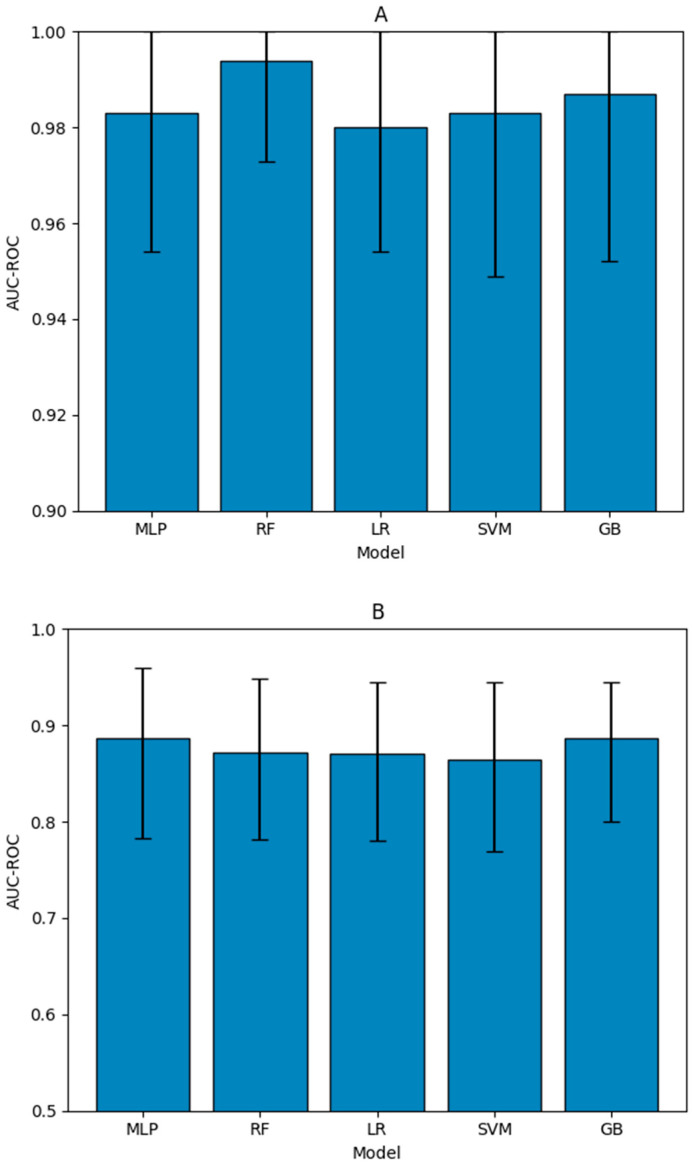
AUC-ROC of the different models in 5-fold cross-validation on the initial training set (**A**) and the independent testing set (**B**). AUC-ROC is the area under the ROC curve, which is a graphical representation of a model’s performance at various classification thresholds; AUC-ROC quantifies the overall ability of the model to distinguish between the positive and negative cases. Training confidence intervals are obtained through repeated stratified 5-fold cross-validation, performed ten times, and the middle 95% of the results are used. The testing confidence intervals are obtained through bootstrapping.

Next, we built several ML models using our training set. All models demonstrated strong and acceptable performance in cross-validation (Figure 2). Therefore, we selected a model with good overall performance, specifically the multi-layer perceptron, which had the second-highest AUC-ROC lower confidence bound (all upper confidence bounds were at 1) and a higher mean than logistic regression, which was equal in terms of lower confidence bounds. The random forest model was not selected due to extremely poor performance when testing on just the independent PASC descriptors after cross-validated training, indicating likely overfitting.

**Figure 3:**
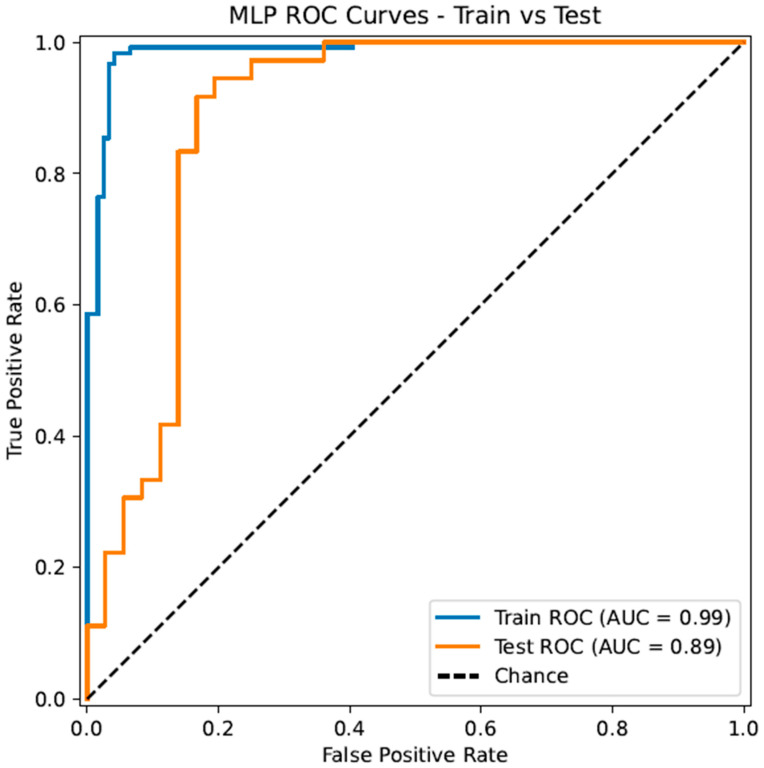
Receiver operating characteristic (ROC) curves for the training set and independent testing set. ROC curves demonstrate the tradeoff between true and false positive rates at various classification thresholds.

For further investigation, we visualize the ROC curves of the training and testing sets side by side. The ROC curves shown in Figure 3, with consistently high and increasing performance over threshold changes, demonstrate that, indeed, a molecular descriptors-based approach with Weka’s feature selection algorithm is conducive to predictive data and robust machine learning algorithms over all classification thresholds. Additionally, proficient testing results, which accompany training results, demonstrate the model’s ability to generalize to unseen datasets.

**Figure 4:**
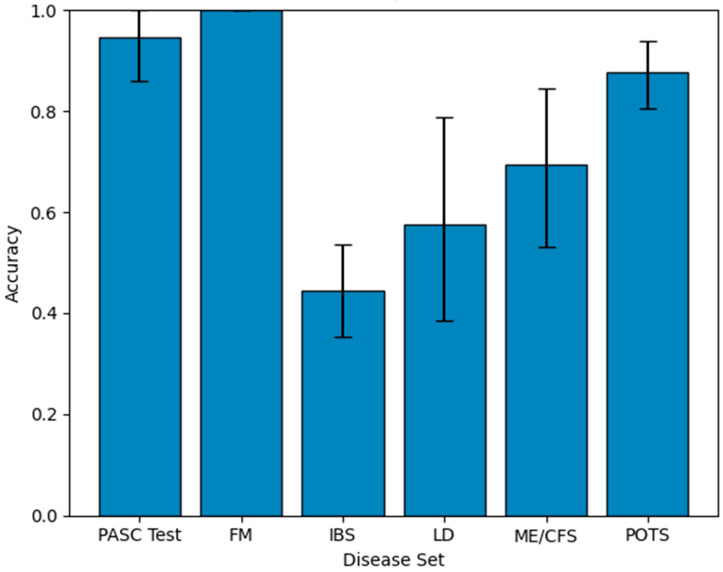
Mean accuracy and bootstrapped confidence intervals of identification based on the percent of dysregulated metabolites of PASC-similar disease classified as PASC by the ML model trained to recognize PASC as the dysregulation source using molecular descriptors of dysregulated metabolites.

With each of these models, we further tested them against dysregulated metabolite sets from an independent PASC set, as well as other diseases often confused with PASC due to similar symptomology: FM [28], Lyme disease [29], ME/CFS [30], POTS [31], and IBS [32]. For testing on independent sets, we bootstrapped the testing sets for five hundred samples, returning the mean and 95% confidence interval for each, in order to clarify in the impact of stochastic variation. The results of this testing are demonstrated in Figure 4.

Observing this graph, we see that the PASC model classifies the dysregulated metabolites of different diseases in a way that confidently separates PASC from Lyme disease, ME/CFS, and IBS. It is able to weakly separate PASC from POTS, with the average PASC classification exceeding that of POTS. However, the 95% confidence intervals show a sizeable overlap between the lower PASC interval and the upper POTS interval. Finally, we chose a threshold, focusing on the proportion of POTS metabolites classified as PASC, as POTS was the most challenging to differentiate from PASC, with the highest proportion of dysregulated metabolites classified as PASC, excluding FM, which none of the models can differentiate from PASC. We lower the confidence level until the PASC and POTS intervals are disjointed, and we find that this occurs at a confidence level of 0.868 and a classification proportion of 0.912. This threshold clearly differentiates PASC from all other PASC-similar diseases, except POTS, which can be differentiated from with weak confidence, and FM.

**Figure 5:**
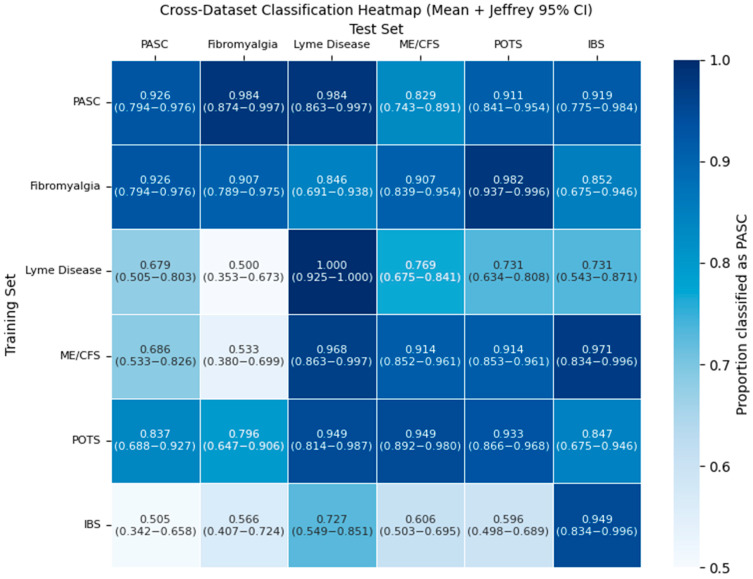
Proportion of dysregulated metabolite training sets for each PASC-similar disease (columns) that are classified as positive by the ML model trained to recognize if each other’s PASC-similar disease (rows) is the dysregulation source using molecular descriptors of dysregulated metabolites. A gradient going from white to blue indicates the relative magnitude of the positive classification proportion. Confidence intervals are 95% Wilson intervals.

To better understand how the molecular descriptors of the metabolites are dysregulated in each of the PASC-similar diseases, we trained a model on the training set of each disease and tested it on the training sets of the other diseases. To ensure consistent results, all models used the same MLP model and training pipeline as the main PASC model. Classification proportions for diseases are shown in Figure 5.

The classification rate for many diseases is high, reaching at least 0.9 classification proportion. However, this is only the case for a few pairs of PASC-similar diseases. One example of unidirectional is with Lyme disease and PASC. While the Lyme disease model easily mistakes many of PASC’s dysregulated metabolites as Lyme disease at a proportion of 0.9837, PASC only classifies 0.6786 of the dysregulated metabolites of Lyme disease as PASC.

## 4. Discussion

We found that molecular descriptors of each plasma metabolite provide sufficient information to classify, with high accuracy, whether it is dysregulated by long COVID. This is evidenced by many machine learning models performing well using 5-fold cross-validation on the training sets, with an average AUC-ROC of up to 98.37%. The ability of molecular descriptors to predict a relationship with PASC can be generalized beyond the training set, as demonstrated by an AUC-ROC of 87.7% in our independent testing set. In addition to being able to separate PASC patients from healthy individuals, our results show that molecular descriptors of dysregulated metabolites are sufficient to differentiate between patients with PASC and PASC-similar diseases, a task that is very challenging in traditional diagnoses without exclusion. For Lyme disease, ME/CFS, and POTS, this is by a large margin.

A significant anomaly in our study was the inability of this approach to separate FM and PASC. The PASC models consistently classified FM as PASC at a rate as high or higher than they classified the independent PASC testing set. This strong overlap is further emphasized by the results of the pairwise classification task. The heatmap demonstrated a uniquely strong overlap between FM and PASC classifications, as one of the only two pairs of PASC-similar diseases where both models classify the other disease’s dysregulated metabolites as its own with a proportion greater than 0.9, indicating an especially strong connection in terms of molecular descriptors between these pairs of diseases. What is most notable is that PASC and all diseases with PASC-similar symptoms, besides FM, have a viral origin and are distinguishable by our set of ML models. FM is a central sensitization disorder with neuroendocrine, mitochondrial, and genetic contributions [37,38,39,40], even though both conditions involve mitochondrial dysfunction [41,42,43,44,45].

This result reinforces and aligns with the existing literature, which supports a connection between the two diseases. FM and PASC are known to exhibit many of the same pathophysiological mechanisms, including autonomic nervous system dysfunction and immune dysregulation [46]. Indeed, these shared manifestations have a significant effect on diagnosis—independent cohorts of PASC patients found 20 to 70% of patients met diagnostic criteria for fibromyalgia, indicating significant overlap within the symptoms of these two conditions [12,37]. Both conditions are associated with low-grade immune activation and persistent inflammation, accompanied by elevated pro-inflammatory cytokines interleukin-6 and tumor necrosis factor [47].

It remains to be seen whether molecular descriptors can distinguish the metabolites dysregulated by these two diseases or if the metabolites dysregulated by these diseases are too similar. In future research, it may be worthwhile to explore further dysregulated metabolite sets for PASC and FM to more confidently ascertain if this approach can distinguish PASC from FM. Additionally, combining this ML/metabolites-based approach with other diagnostic methods, such as transcriptomics, proteomics, and clinical symptoms, may reveal an additional diagnostic strength. It also advised investigating other biomarkers, for example, small non-coding RNAs.

## 5. Limitations

One significant limitation of this study is that our analysis and selection of a threshold depend heavily on the datasets themselves, which may vary due to demographic differences. Most significantly, there is a nontrivial confounder for age between the training and testing datasets, with the training dataset having noticeably older patients, and both datasets having older PASC cohorts than healthy controls. Because the papers from which the datasets were drawn do not provide demographic data paired with biological data, it is difficult to identify the true confounding effect of age. There may have been other confounders within the data, but complete demographics are not provided by the papers from which the data is sourced.

Before applying our work in a clinical context, more datasets should be used for training and testing to reduce the impact of chance variance in demographics. Working with more sets encompassing diverse demographics may better validate the robust generalizability of our model and the threshold that we defined for classifying the whole set as PASC.

## 6. Conclusions

A critical roadblock in PASC diagnosis is the reliance on exclusion, stemming from its lack of distinctive biomarkers, which makes diagnosis challenging and time-consuming. We hypothesized that we could remediate this issue by utilizing the molecular descriptors of dysregulated metabolites in PASC patients to create a machine-learning model that can identify PASC-associated metabolic dysregulation and support the differentiation between PASC and PASC-like conditions based on metabolomics profiles.

Our findings support this hypothesis: we were able to create a model that not only differentiates PASC from healthy controls, but also successfully differentiates PASC from several similar conditions. In principle, our approach to PASC diagnosis is assay-agnostic and should offer because input descriptors can be generated regardless of the metabolite measurement assay used. Yet, the datasets used to train our model from Wang and López-Hernández et al. exclusively used LC-MS/MS and FIA-MS/MS metabolite assays. Thus, validation with further datasets is imperative to evaluate this method’s potential for generalizability.

Our model demonstrates the feasibility of automating the exclusion of multiple PASC-similar diseases, representing a novel step in PASC diagnosis. Refinements of this process could be used in conjunction with other techniques to alleviate the burdens of manual exclusion processes and enhance the efficiency and accuracy of diagnoses. Ultimately, diagnostic improvements could lead to earlier interventions and better outcomes for patients with PASC.

Beyond demonstrating that a molecular descriptors-based approach allows us to automatically distinguish PASC from many PASC-similar diseases, our study found a unique connection between PASC and FM: PASC and FM consistently overlapped in their classification by descriptors-based machine learning models, despite the fact that they have different origins, even more so than other PASC-similar diseases. This uniquely strong overlap suggests that the two diseases are strongly intertwined at a molecular level, reinforcing the literature that supports shared mechanisms and symptomology. Ultimately, this highlights fibromyalgia as a key comparator in PASC-related studies and vice versa.

## Figures and Tables

**Table 1:** Quantitative data is presented as median (Q1–Q3) and qualitative data is presented as *n* (%). The papers do not provide other forms of demographic data comparing PASC and healthy controls.

Characteristic	PASC Training (*n* = 117)	Healthy Training (*n* = 28)	PASC Testing (*n* = 48)	Healthy Testing (*n* = 37)
Age (years)	62 (53–73)	55 (52–59)	51.5 (43.5–60.8)	40.5 (37–53.3)
Male	66 (56.4)	16 (57.1)	28 (58.3)	17 (44.7)

## Data Availability

The original contributions presented in this study are included in the article and Supplementary Materials (archived on Github at https://github.com/Ethanc143/Metabolomics-Based-Machine-Learning-Diagnostics-of-Post-Acute-Sequelae-of-SARS-CoV-2-Infection, 10 November 2025). Further inquiries can be directed to the corresponding author.

## Supplementary Materials

The following supporting information can be downloaded at: https://github.com/Ethanc143/Metabolomics-Based-Machine-Learning-Diagnostics-of-Post-Acute-Sequelae-of-SARS-CoV-2-Infection (accessed on 10 November 2025).

## Abbreviations

API	application programming interface
AUC	area under curve
CACTUS	CADD Group Chemoinformatic Tools and User Services
CADD	computer-aided design and drafting
FM	fibromyalgia
HMDB	Human Metabolite Data Base
IBS	irritable bowel syndrome
LMT	logistic model tree
ME/CFS	myalgic encephalomyelitis/chronic fatigue syndrome
ML	machine learning
MLP	multi-layer perceptron
NCI	National Cancer Institute
PASC	Post-Acute Sequelae of COVID-19
PCC	post-COVID-19 condition
PCS	post-COVID-19 syndrome
POTS	postural orthostatic tachycardia syndrome
PR	precision–recall
RECOVER	Researching COVID to Enhance Recovery
ROC	receiver operating characteristic
SGD	stochastic gradient descent
SMILES	Simplified Molecular Input Line Entry System
SMO	sequential minimal optimization
SVM	support vector machine
WEKA	Waikato Environment for Knowledge Analysis
WHO	World Health Organization
